# Practical Points

**Published:** 1892-04-30

**Authors:** 


### PRACTICAL POINTS.

Questions are invited for this column on any point in the administration of hospitals and asylums which may prove of difficulty or interest to our readers. We propose to give a few typical questions and answers, and shall continue them from time to time providing the idea is taken up by those for whose benefit it is chiefly intended. All communications should be marked "Practical Points," addressed to the Editor of The Hospital, 140, Strand, W.C.

*Convalescent Home Construction.*—"*Hospital Secretary*" *asks: Can you inform me where I can obtain plans of recently-erected convalescent homes?*

Will any architect reply to this correspondent?

*London's Provision for Paying Patients.*—"*Claremont*" *writes: Will you give me some information as regards paying patients in a London home?*

There are several pay hospitals now in London. The original of these is the Home Hospital in Fitzroy Square; paying patients are also received at St. Thomas's Hospital, Bollingbrooke House pay hospital, Clapham Common; and at the Hampstead Home Hospital, Parliament Hill Road, N.W.

